# Obesity and Chronic Inflammation: Implications for Rheumatoid Arthritis, Spondyloarthritis, and Ulcerative Colitis

**DOI:** 10.1002/iid3.70080

**Published:** 2025-01-06

**Authors:** Ada Corrado, Ilaria Guadagni, Giovanna Picarelli, Angela Variola

**Affiliations:** ^1^ Department of Medical and Surgical Sciences, Rheumatology Clinic University of Foggia Foggia Italy; ^2^ Medical Department Pfizer Italy Rome Italy; ^3^ IBD Unit IRCCS Sacro Cuore Don Calabria Negrar Italy

**Keywords:** chronic inflammation, immune‐mediated inflammatory diseases (IMIDs), inflammatory bowel disease (IBD), obesity, rheumatoid arthritis (RA), spondyloarthritis (SpA), ulcerative colitis (UC)

## Abstract

**Background:**

Immune‐mediated inflammatory diseases (IMIDs) are a group of chronic conditions characterized by dysregulated immune responses and persistent inflammation. Rheumatoid arthritis (RA), spondyloarthritis (SpA), and ulcerative colitis (UC) exemplify prominent IMIDs, each presenting unique challenges for their management, that impact patient's quality of life (QoL). Obesity, marked by persistent low‐grade inflammation, influences the progression, response to treatment, and clinical management of patients with RA, SpA, and UC. Besides, the emerging role of sarcopenic obesity, a special subtype of obesity with malnutrition, should be considered in the definition of the appropriated therapeutic interventions.

**Methods:**

This narrative literature review summarizes recent evidence on the interplay between obesity‐induced inflammation and IMIDs.

**Results:**

Obesity contributes to elevated levels of proinflammatory cytokines, influencing the inflammatory pathways common to IMIDs. White adipose tissue, acting as an endocrine organ, produces cytokines like TNF‐α and IL‐6, fueling chronic inflammation. The dysregulation of adipokines, such as leptin and adiponectin, further complicates this interplay, impacting immune responses and metabolic processes.

**Conclusions:**

Understanding the cross‐talk between inflammatory pathways in obesity and IMIDs can provide insight into potential targets for intervention. This includes lifestyle modifications aimed to regulate weight gain, paving the way for comprehensive strategies to manage IMIDs in the context of obesity.

## Graphical abstract

The cross‐talk between adiposity‐driven inflammation and immune‐mediated inflammatory diseases (IMIDs) underscores the systemic impact of obesity on the exacerbation of IMIDs. Sarcopenic obesity, characterized by the coexistence of muscle mass loss and excessive fat accumulation, further complicates the clinical management and outcomes of IMID patients. Addressing obesity through lifestyle changes and potentially adjusting therapeutic strategies could significantly improve disease outcomes, quality of life, and the efficacy of pharmacological treatments for patients with IMIDs.

## 1 Introduction

Immune‐mediated inflammatory diseases (IMIDs) encompass a range of persistent and disabling conditions with diverse clinical manifestations, etiologies, and organ involvement. These diseases share an underlying immune system's dysregulation, resulting in unprovoked chronic inflammation across multiple organs and tissues. Moreover, IMIDs often present a series of comorbidities, that can be considered as clinical manifestations of the underlying systemic inflammation, such as metabolic issues and cardiovascular diseases. Such comorbidities complicate IMID management and impair patient's quality of life (QoL) [1]. Among IMIDs, prominent examples are musculoskeletal diseases such as rheumatoid arthritis (RA) and spondyloarthritis (SpA), and inflammatory bowel diseases (IBD) such as ulcerative colitis (UC) and Crohn's disease (CD), that are posing unique challenges to patients and healthcare providers.

RA is a systemic chronic autoimmune disease characterized by chronic inflammation of the synovial joints, leading to progressive joint destruction and extra‐articular manifestations [2].

SpA defines a group of pathological conditions including axial SpA and psoriatic arthritis (PsA) with similar clinical features such as involvement of axial skeleton and a distinctive pattern of peripheral joint involvement, characterized by enthesitis and asymmetric oligoarthritis, often accompanied by extra‐articular manifestations [3].

UC is a chronic form of IBD affecting the mucosa of the rectum and colon. IBDs include also CD and unclassified colitis, all involving inflammation of gastrointestinal tract, leading to abdominal pain, diarrhea, rectal bleeding, fatigue, weight loss, and other potential extra‐intestinal complications [4].

While the prevalence and incidence vary by geographic region and population demographics, the overall impact of these diseases is substantial on global healthcare systems. Estimated prevalence is between 0.5% and 1.35% for RA and SpA [5, 6], while for UC is around 0.06% with a significant increase worldwide [4]. Prevalence of UC is around double compared to CD in Europe [7].

Among metabolic comorbidities related to IMIDs, obesity stands out as a significant concern due to its association with chronic low‐grade inflammation and immune system activation, that in turn, may impact the disease course of RA, SpA, and UC. Characterized by excessive accumulation of adipose tissue, obesity affects individuals of all ages, genders, and socioeconomic backgrounds, and it has dramatically increased worldwide over the past 30 years [8]. The body mass index (BMI), which calculates a person's weight in relation to their height, is the most common measure to classify obesity. In particular, a BMI ≥ 30 kg/m² classifies an individual as obese, while a BMI between 25 and 30 kg/m² categorizes a person as overweight [9]. Obesity precipitates a cascade of metabolic issues such as cardiovascular risk, including insulin resistance, dyslipidemia, and diabetes, which collectively contribute to the pathogenesis of many other health complications [10, 11].

Various studies have consistently reported a notable proportion of IMID patients exhibiting obesity, with prevalence rates hovering just above 20% [12, 13, 14].

In this review we aim to elucidate the ways in which obesity impacts and influences the medical management of patients with IMIDs, to identify potential comprehensive approaches. We focused our analysis on the musculoskeletal conditions RA and SpA, and among the IBDs, we mainly focused on UC, given the higher prevalence of the latter compared to CD [7], especially in the Italian context [15].

## 2 Methods

We searched PubMed with the terms (“Rheumatoid Arthritis” OR “Spondyloarthritis” OR “Ulcerative Colitis” OR “inflammatory bowel disease”) AND “Obesity,” within the time window 2010−2023. During the selection process, articles published in the time frame 2018−2023 were prioritized. Authors independently reviewed the outcome of the research and selected the papers to be discussed according to their background (rheumatology or gastroenterology).

## 3 Inflammatory Pathways in IMIDs and Obesity

The chronic inflammation of RA, SpA, and UC involves several cytokines and signaling pathways that are also involved with the systemic inflammation associated with obesity.

Prominent proinflammatory cytokines involved in the pathogenesis of RA, SpA, and UC are TNF‐α and interleukin (IL)‐6 [16]. Various joint cell types produce and respond to TNF‐α, contributing to chronic tissue damage. TNF‐α also initiates and perpetuates proinflammatory processes within the intestinal mucosa in UC, facilitating the recruitment of inflammatory cells and stimulating other proinflammatory cytokines for tissue remodeling [16].

IL‐6 stimulates acute‐phase mediators and contributes to joint inflammation and cartilage destruction, influencing leukocyte infiltration and regulating B lymphocyte activity. IL‐6 levels are also elevated in the intestinal mucosa of IBD individuals [16].

Other prominent cytokines involved in IBD inflammation are IL‐12 and IFN‐γ [16]. In SpA, COX‐2 activation leads to IL‐17A overproduction, a principal cytokine involved with the disease [16]. IL‐17/IL‐23 axis stimulation is common in SpA and IBD, linking the two diseases [17]. In particular, in IBD, IL‐17A has a protective role in the gastrointestinal tract, while IL‐23 perturbs the balance between proinflammatory and anti‐inflammatory pathways, triggering colitis without systemic inflammation [18, 19]. Several effects of these cytokines are mediated by the JAK‐STAT signaling pathway, making its inhibition an effective treatment tool in all IMIDs [16].

Structural cells within tissues, constituting the microenvironment for cytokine signaling, can actively modulate the differential responses to inflammatory stimuli in IMIDs [20]. For instance, the production of IL‐17F by immune cells is enhanced by the interaction with synoviocytes in RA, emphasizing the heterogeneous and cell‐specific evolution of each inflammatory disease [20]. Indeed, the tissue‐specific functions of IL‐17 and IL‐23 highlight how alternative signaling pathways can be involved in perpetuating inflammation in the different IMIDs [21].

Chronic inflammation in obesity is sustained by the role of white adipose tissue as a producer of cytokines [22]. White adipose tissue acts not merely as an energy storage component but as an active endocrine organ. In fact, besides adipocytes, white adipose tissue is composed of immune cells. The interplay between adipocytes and immune cells modulates their secretion pattern, impacting metabolic function and inflammation levels in the body. In lean individuals, immune cells within white adipose tissue present an anti‐inflammatory phenotype, including M2‐macrophages and regulatory T cells, that maintain healthy metabolic homeostasis through the secretion of anti‐inflammatory cytokines like IL‐4, IL‐10, and IL‐13 [22]. In obese individuals, hypertrophy of lipid‐loaded adipocytes is accompanied by a change in the phenotype of associated immune cells, with macrophages acquiring a specific metabolically active proinflammatory phenotype [22], and the infiltration of other proinflammatory immune cells such as IL‐17‐producing T (Th17) cells [23]. Thus, white adipose tissue in obesity secretes a spectrum of proinflammatory molecules, including IL‐6, IL‐8, IL‐17, IL‐23, monocyte chemoattractant protein‐1 (MCP‐1), plasminogen activator inhibitor 1 (PAI‐1), establishing systemic obesity's chronic low‐grade inflammation. Indeed, obese patients have shown increased levels of circulating IL‐17A and IL‐23, which are prominent cytokines involved in inflammatory diseases [23].

In addition, white adipose tissue secretes a specific set of cytokines known as adipokines, with a wide range of functions not yet completely understood, spanning from modulation of physiological functions to metabolic and inflammatory pathways [22]. Among adipokines, leptin acts as an indicator of energy reserves, regulating long‐term energy balance and body fat levels. By binding to its receptor within the hypothalamic feeding center, in physiological conditions, leptin regulates feeding behavior to maintain long‐term normal body weight. Notably, one of the signaling pathways involved in leptin's physiological control of energy is the JAK2/STAT3 [24]. Interestingly, a potential correlation between inflammatory cytokine IL‐17A and leptin increase in human bone marrow mesenchymal stem cells in vitro has been reported [25].

Obesity is characterized by enhanced production of leptin accompanied by resistance to its signaling, resulting in the disruption of the body's energy balance [26]. In addition, leptin has been shown to stimulate the inflammatory phenotype of T‐cells, macrophages, and other innate immunity cells, which in turn can fuel the production of proinflammatory mediators such as TNF‐α and IL‐6, both acting through the JAK/STAT pathway. This results in increased inflammatory levels and immune responses that sustain immune‐mediated diseases [22].

Adiponectin, another prominent adipokine, enhances insulin sensitivity in major insulin target tissues, contributing to the regulation of glucose levels and fatty acid breakdown [22]. Adiponectin exerts a protective role against inflammation; however, conflicting evidence exists concerning its action. While obesity is associated with reduced levels of adiponectin and increased content of inflammatory macrophages in adipose tissue, in some immunoinflammatory diseases such as RA, increased adiponectin levels are associated with a concomitant increase of IL‐6 [27]. The different adiponectin isoforms and/or the different sources of its generation (white adipose tissue from lean or obese individuals) may play a role in its final effect on immune cells [22].

Therefore, adipokines should be regarded for their systemic role in modulating inflammation throughout the body. This underlines their role as a potential tool to modulate inflammatory pathways.

Another type of adipose tissue present in the human body is brown adipose tissue, involved in energy expenditure and thermogenesis [28]. Recent research has focused on the possible role of brown adipose tissue in the modulation of inflammation and obesity, and some reports showed its enhanced activity and increased presence in lean individuals compare to obese individuals, pointing at a possible protective anti‐inflammatory role [28].

## 4 Obesity Modulates Disease Progression in IMIDs

### 4.1 Obesity in RA and SpA

Obesity has been associated with greater arthritis activity, worse overall disease course, less frequent remissions, and a reduced probability of response to therapies [29, 30]. A prospective, comparative, cross‐sectional study performed on RA patients assessed the association between increased weight and clinical RA status [31]. High BMI was associated to increased disease activity in RA subjects as evidenced by a greater number of swollen joints and a worsen disease course [31]. In the QUEST‐RA study, obese RA patients had a higher mean Disease Activity Score 28 (DAS28) compared to normal‐weight patients [32]. Furthermore, a registry‐based study found that obesity is linked to heightened pain levels and augmented incidence of distress symptoms in individuals with RA, thus impacting QoL besides clinical aspects [33].

In SpA as well, obesity was proven to be an independent predictor of poor clinical outcomes and reduced response to treatment, significantly correlating with longer symptom duration, higher disease activity, worse physical function, and an overall deterioration of QoL [29, 34].

Despite the evidence supporting the association between obesity and disease worsening, the specific molecular mechanisms involved are still unclear.

### 4.2 Obesity in UC

Epidemiological data reveal a notable increase in the prevalence of obesity among individuals with IBD, highlighting the need for monitoring the characteristics of this specific subset of patients [14].

Obesity has been observed to correlate differently with the disease course of UC and CD. While a study showed reduced rates of disease remission and an increased risk of experiencing a complex disease course in obese patients with CD but not UC [35], another research, focused on patient‐reported outcomes, underscored a similar elevated risk of experiencing persistent disease activity or relapse in both UC and CD obese populations [36]. Although obesity appears not to be associated with increased hospitalization, surgery, or serious infections in a multicenter cohort of patients with IBD [37], among hospitalized IBD patients, those who were obese experienced longer hospital stays and more complications compared to nonobese patients [38].

Response to treatment is also impaired in obese IBD patients, with an association of elevated treatment failure with BMI increase in UC [39], and increased request of drug dose escalation [40].

### 4.3 Sarcopenia and Sarcopenic Obesity

Sarcopenia is a condition characterized by progressive skeletal muscle deterioration, muscle mass, and function loss, frequently accompanied by a substantial excessive accumulation of visceral fat and an overall increase in body weight. The latter manifestation is known as sarcopenic obesity [41]. Sarcopenic obesity can play a role in the progression of IMIDs, as the loss of muscle mass combined with increased adipose tissue exacerbates immune dysregulation [42].

Sarcopenic obesity is described in RA, with a prevalence in elderly and women, and IBD patients [43, 44, 45]. There is a scarcity of research specifically focused on SpA, although a recent systematic review analyzed sarcopenia in ankylosing spondylitis and supported an association between the disease and muscle health deterioration, particularly muscle strength, even in young patients [46].

In IBD, sarcopenic obesity has been linked to poor prognosis and worse clinical outcomes, with higher likeliness to undergo surgery or rehospitalization and with high rates of post‐surgery complications [43]. A recent work analyzing studies on IBD patients highlighted the need for a standardized evaluation of sarcopenia in this population, which could lead to a better evaluation of the risk for surgery risk and poor outcomes [47]. The need for nutritional screening and assessment in IBD patients may be a possible point of further discussion among the scientific community.

## 5 Conventional and Advanced Therapies for Disease Activity Modulation in the Context of Obesity

IMIDs are pharmacologically treated with conventional and advanced therapies. Managing IMIDs in obese patients can be challenging in clinical practice, since obesity‐related inflammatory pathways may complicate treatment and potentially diminish therapy effectiveness [40]. Obesity status can specifically alter the pharmacokinetics and, consequently, the drug availability at the target site of action, leading to reduced efficacy [40]. Increased volume of distribution and faster drug clearance are proposed mechanisms associated with obesity resulting in lower plasma drug concentration [40]. A clear consensus regarding the preferred administration route (oral, subcutaneous, and intravenous therapy), dosing scheme (weight‐based vs. fixed dosing), or the preferred mechanism of action (e.g., TNF inhibitors vs. IL inhibitors) of pharmacological treatments for obese patients with IMIDs remains elusive.

### 5.1 Impact of Obesity on Treatment Efficacy in RA and SpA

In the comprehensive management of rheumatological diseases, treatment for RA and SpA involves disease‐modifying antirheumatic drugs (DMARDs), with traditional options like methotrexate, biologic DMARDs (such as TNF‐inhibitors, anti‐IL‐6 in RA, and anti‐IL17/23 in SpA) and targeted synthetic (ts) DMARDs (such as JAK inhibitors) designed to modify the course of the disease [48, 49]; nonsteroidal anti‐inflammatory drugs (NSAIDs) and corticosteroids to provide symptomatic relief.

Several data highlight that obesity may influence the effectiveness of therapies in managing RA patients, with reduced chances of achieving low disease activity [50, 51]. In particular, it has been shown that obese patients fail to achieve a low disease activity upon 6‐month treatment with TNF‐inhibitors (35.6% obese patients in the low disease activity group and 72% in the non‐low disease activity group, *p* = 0.004), although no correlation was observed in patients treated with an anti‐IL‐6, hinting that the response to the latter therapy may be independent of obesity [51]. Moreover, the study showed that leptin levels were significantly higher after 6 months of treatment with a TNF inhibitor in patients not reaching low disease activity, whereas no difference was observed in adiponectin levels [51]. No difference in either leptin or adiponectin was instead observed upon anti‐IL‐6 treatment [51]. Another study showed a direct association between disease activity in RA patients and leptin levels, while an inverse relationship was seen for adiponectin [52]. Indeed, TNF inhibitors reduced leptin and increased adiponectin serum levels in RA, supporting the fact that a possible action on disease activity may go through the modulation of adipokine levels [52]. Nevertheless, the detailed relationship is yet to be elucidated, as well as the reason why this response occurs only in some individuals [51]. A recent prospective observational study from the Nordic Rheumatic Diseases Strategy Trials and Registries showed that obesity was associated to a reduced effectiveness of treatments for RA, including biologic DMARDs such as selective T‐cell co‐stimulation modulators and IL‐6 inhibitors [53]. This emphasizes the complexity of the mechanism implicated in RA treatment in obese patients, which requires more longitudinal studies to fully understand the impact of obesity on long‐term RA outcomes.

Treatment response to TNF inhibitors was also impacted in overweight and obese patients with ankylosing spondylitis [54, 55].

Conflicting evidence is also present when considering the use of advanced treatments such as JAK inhibitors. A pooled analysis from six Phase 3 studies investigating the efficacy of a JAK1/JAK3 inhibitor in patients with RA revealed no differences in efficacy outcomes across BMI categories (BMI < 25, 25 to < 30, and ≥ 30 kg/m²). Response to the JAK1/JAK3 inhibitor remained consistent regardless of baseline BMI, indicating that this treatment option is effective for individuals with moderate to severe RA, including those with a BMI ≥ 30 kg/m² [56]. Similarly, a specific JAK1 inhibitor showed the same efficacy in patients with early RA when considering BMI subgroups (BMI < 25 and BMI ≥ 25) [57]. A study showed that in PsA, the efficacy of a JAK1 inhibitor was independent of BMI [58]. Conversely, pooled data from two Phase 3 studies, involving 710 patients with PsA, with 43.8% of obese (BMI ≥ 30 kg/m²), showed a reduction in JAK1/JAK3 inhibitor efficacy in patients with a baseline BMI ≥ 35 kg/m² [59].

When considering anti‐IL‐17 or anti‐IL‐23 treatment, the effects of overweight and obesity on treatment efficacy are less defined. Overall, a lower number of studies are currently available discussing the impact of obesity on anti‐IL‐17 or anti‐IL‐23 with respect to TNF inhibitors, and the differences in response between obese and nonobese patients resulted less prominent [60]. Limited data are available on anti‐IL‐17 in SpA. A preliminary report on patients with ankylosing spondylitis treated with anti‐IL‐17 indicates an improvement in disease activity irrespective of BMI [61]. More recently, an observational cohort study from Turkey reported no significant differences in both treatment response and anti‐IL‐17 therapy continuation between obese and nonobese ankylosing spondylitis patients [62]. Nevertheless, further data are required to better characterize the response to these agents in this patient population.

### 5.2 Impact of Obesity on Treatment Efficacy in UC and CD

The therapeutic approach for UC involves 5‐aminosalicylates, corticosteroids, thiopurines, biologics (TNF‐inhibitors, IL inhibitors, anti‐integrins), and targeted small molecules such as JAK inhibitors [63].

A recent meta‐analysis revealed that obese patients with UC faced a higher risk of TNF‐inhibitor treatment failure compared to nonobese counterparts, highlighting a 19.5% higher odds of treatment ineffectiveness, regardless of whether patients received fixed‐dose or weight‐based treatment regimens [64]. Notably, this association was not observed in patients with CD [64]. Higher treatment failure can be a consequence of the adipose tissue's role as an endocrine organ influencing inflammatory processes. Moreover, the differential impact of obesity on UC versus CD patients suggests that the pathophysiology of obesity's effect on IBD may be more complex, particularly considering the unique role of mesenteric fat in CD [64].

Few data are available on the impact of BMI on the use of JAK inhibitors in individuals with IBD. A recent study demonstrated that the efficacy and safety profile of a JAK1/JAK3 inhibitor in patients with UC remained consistent regardless of variations in baseline BMI [65]. This finding hints at the potential use of JAK inhibitors as a viable treatment option for UC patients despite obesity status. Moving forward, additional research into the interactions between obesity, treatment efficacy, and the pathophysiology of IBD is required to develop more effective personalized treatment strategies in this patient subgroup.

### 5.3 Addressing Obesity in IMIDs

Similarly to other diseases also in IMID drug therapy efficacy decreases in obese patients. Correcting obesity may become a central step for the management of these patients [66].

Indeed, obesity is a modifiable factor, meaning that it can be changed or managed through various approaches with benefits reaching not only the individual's weight and well‐being but also inducing potential improvements in the course of concomitant diseases. Lifestyle changes targeting weight loss, such as ameliorating diet, increasing physical activity, and behavior counseling, are nonpharmacological options to improve disease activity in obese patients [67]. Weight loss interventions can lead to decreased production of inflammatory mediators by adipose tissue, thereby dampening overall chronic body inflammation. Moreover, weight loss can contribute to improved QoL of IMID patients who may experience reduced fatigue, improved mobility, and psychological well‐being [68]. A randomized controlled trial on 40 obese RA patients who underwent a hypocaloric diet intervention showed that patients who successfully achieved weight loss reached significant improvements in RA disease activity markers and patient‐reported outcomes compared to the control group. Interestingly, serum leptin levels decreased while adiponectin increased, suggesting a biological response to weight loss that could influence RA disease activity [69]. A cross‐sectional study analyzing the relationship between the nutritional status and dietary intake of RA patients and the disease activity also supports the influence of nutritional intervention on RA course. The study found a significant negative correlation between the dietary intake of certain nutrients (e.g., omega‐3 fatty acids, calcium) and disease activity. On the other hand, omega‐6 fatty acids were positively associated with inflammation and increased disease activity. The modification of the diet of RA patients, especially concerning anti‐inflammatory nutrients, could modulate disease activity and improve treatment outcomes, suggesting that dietary interventions could complement pharmacological treatments [70].

This approach aims to enhance also the response to pharmacological treatment, and may positively influence other obesity‐related comorbidities which are common among individuals with IMIDs, such as the increased cardiovascular disease risk profile [11, 71], with the establishment of a virtuous circle that aims to the reduced inflammation and improve the disease management.

Indeed, data from a case series of patients with RA and PsA who lost > 10% of body mass experienced a decrease in disease activity and a substantial improved disease remission (from 6% to 63% of patients) without changes in the pharmacological therapy [71].

On the other hand, pharmacological interventions directed at the JAK‐STAT pathway have shown promise in addressing metabolic issues, such as mitigating insulin resistance [72]. The metabolic activation of white adipocytes toward a brown adipose tissue phenotype is the possible connection between pharmacological and metabolic control of inflammation [72, 73]. The modulation of adipose tissue composition may further influence obesity and, in turn, also the disease activity sustaining the overall improvement. A comprehensive exploration of the reciprocal relationship between obesity and IMIDs is essential, emphasizing the need for personalized patient care strategies.

Nonpharmacological intervention such as weight loss is particularly challenging in IBD patients and the evidence of its effect on disease activity and improved response to therapy is limited, underscoring the need for further research and tailored approaches for this patient population [40]. Nevertheless, considering the higher prevalence of sarcopenic obesity in UC patients, increasing the risk of worse prognosis and associated comorbidities [74], a nutritional intervention may be the optimal first line strategy to improve the general state of the patient while addressing the underlying inflammation [75].

To this aim, an initial nutritional screening [76] in UC, but also of RA and SpA patients may help the clinician to define a tailored nonpharmacological intervention and the most appropriated drug therapy, to reach and maintain disease control.

Data concerning baseline nutritional status of RA, SpA, and UC patients are scarce [40, 71] as well as studies analyzing the effect of weight reduction in the long‐term management of IMID patients or in disease remission.

Diet interventions have the potential to impact both disease activity and obesity. American and European Guidelines already include diet recommendations for RA management [77, 78], while a recent systematic review analyzed the existing evidence on diet's influence on SpA [79]. The Mediterranean diet appears to be promising in reducing disease activity in axial SpA, while the evidence for other dietary interventions, such as probiotics and polyunsaturated fatty acids, remains inconclusive [79]. Indeed, patients adherent to the Mediterranean diet showed more frequent improvement in the Ankylosing Spondylitis Disease Activity Score and C‐reactive protein and reported increased regular physical activity, suggesting this diet's potential role in managing inflammation and enhancing clinical outcomes in axial SpA [80].

Lifestyle interventions for obesity typically involve a multidisciplinary team of healthcare professionals, including physicians, dietitians, exercise specialists, nurses, and psychologists [81]. Further intervention studies are needed to better understand the effects of nutritional interventions in IMID patients.

## 6 Conclusions

**Figure 1:**
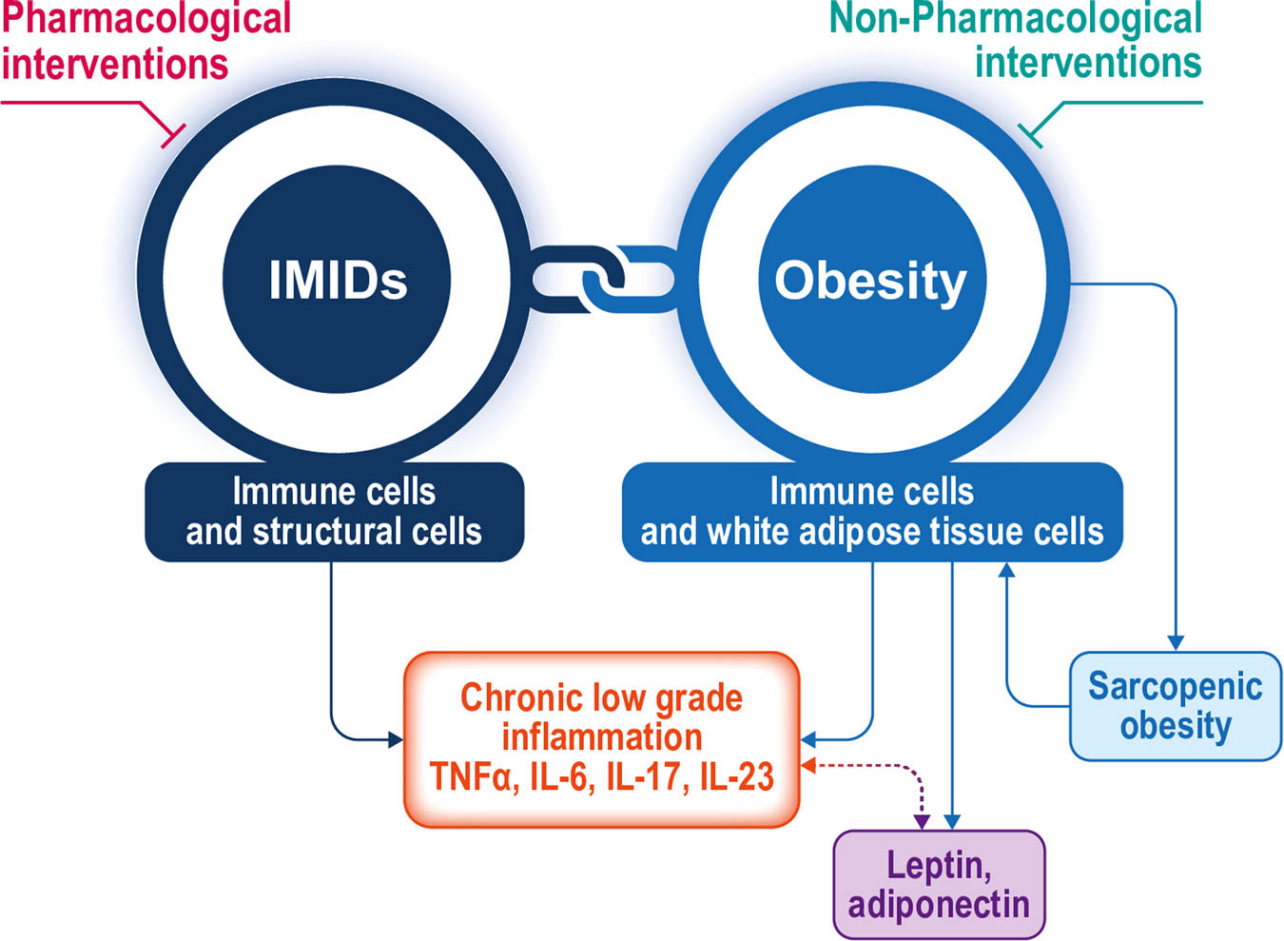
Obesity influence on IMIDs, underlying chronic low‐grade inflammation, and potential interventions.

Obesity influences the course of RA, SpA, and IBD. Chronic inflammation is a hallmark of both obesity and IMIDs, and it is likely that the common active inflammatory pathways may further fuel the development and progression of IMIDs in obese individuals. By fostering a proinflammatory environment and immune imbalance, obesity potentially enhances a worsening of preexisting conditions. Given its modifiable nature, it is of primary importance to target obesity in IMID patients and account for nutritional evaluation to plan early and coordinated interventions that may yield therapeutic benefits for these pathologies (Figure 1).

## Author Contributions

All authors conceived the work, revised the literature, and drafted, reviewed, and edited the manuscript. All authors approved the final version submitted.

## Conflicts of Interest

I.G. and G.P. are Pfizer employees. A.C. and A.V. were paid consultants to Pfizer in connection with the development of this manuscript.

## Data Availability

The authors have nothing to report.
